# Protective effects of hydroponic Teucrium polium on hippocampal neurodegeneration in ovariectomized rats

**DOI:** 10.1186/s12906-016-1407-3

**Published:** 2016-10-24

**Authors:** K. V. Simonyan, V. A. Chavushyan

**Affiliations:** Neuroendocrine Relationships Lab, Orbeli Institute of Physiology NAS RA, Yerevan, 0028 Armenia

**Keywords:** Hippocampus, Spike activity flow, Ca^2+^-dependent acid phosphatase, Ovariectomy, Hydroponic Teucrium polium

## Abstract

**Background:**

The hippocampus is a target of ovarian hormones, and is necessary for memory. Ovarian hormone loss is associated with a progressive reduction in synaptic strength and dendritic spine. Teucrium polium has beneficial effects on learning and memory. However, it remains unknown whether Teucrium polium ameliorates hippocampal cells spike activity and morphological impairments induced by estrogen deficiency.

**Methods:**

In the present study, we investigated the effects of hydroponic Teucrium polium on hippocampal neuronal activity and morpho-histochemistry of bilateral ovariectomized (OVX) rats. Tetanic potentiation or depression with posttetanic potentiation and depression was recorded extracellularly in response to ipsilateral entorhinal cortex high frequency stimulation. In morpho-histochemical study revealing of the activity of Ca^2+^-dependent acid phosphatase was observed. In all groups (sham-operated, sham + Teucrium polium, OVX, OVX + Teucrium polium), most recorded hippocampal neurons at HFS of entorhinal cortex showed TD-PTP responses.

**Results:**

After 8 weeks in OVX group an anomalous evoked spike activity was detected (a high percentage of typical areactive units). In OVX + Teucrium polium group a synaptic activity was revealed, indicating prevention OVX-induced degenerative alterations: balance of types of responses was close to norm and areactive units were not recorded. All recorded neurons in sham + Teucrium polium group were characterized by the highest mean frequency background and poststimulus activity. In OVX+ Teucrium polium group the hippocampal cells had recovered their size and shape in CA1 and CA3 field compared with OVX group where hippocampal cells were characterized by a sharp drop in phosphatase activity and there was a complete lack of processes reaction.

**Conclusion:**

Thus, Teucrium polium reduced OVX-induce neurodegenerative alterations in entorhinal cortex-hippocamp circuitry and facilitated neuronal survival by modulating activity of neurotransmitters and network plasticity.

## Background

Estrogen deprivation is a high risk factor for cognitive dysfunction [1] and recent research has shown estrogen deficiency following ovariectomy (OVX) to have negative effects on learning and memory [2]. Ovariectomy of rats results in a decrease of dendritic spine density in CA1 pyramidal neurons in the hippocampus [3]. It is known that within the hippocampus, both ERα and ERβ localize to dendritic spines, which are sites of synapse formation that show a high degree of plasticity [4] and receptors for estrogen, especially expressed in the CA3 region of hippocampus [5].

The rat, mouse and human estrogen receptor is sensitive to phytoestrogens and while the estrogenic potency of industrial-derived estrogenic chemicals is very limited, the estrogenic potency of phytoestrogens is significant, especially for ER beta and they may trigger many of the biological responses that are evoked by the physiological estrogens. Some phytoestrogens compete stronger with E2 for binding to ER beta than to ER alpha [6]. Phytoestrogens are plant-derived hormone-like diphenolic compounds of dietary origin. These compounds are weakly estrogenic and could play a role in the prevention of other estrogen-related conditions, namely, cardiovascular diseases, menopausal symptoms, postmenopausal osteoporosis, neuroprotective effects [7]. The phytoestrogens are synthesized in plants from phenylpropanoids and simple phenols [8]. Phenylpropanoid glycosides are a relatively new group of active substances, found in many plant species and possessing significant pharmacological potential, related to their antioxidant, neuroprotective, hepatoprotective, immunomodulatory and tyrosinase inhibitory actions and others effects [9, 10]. Molecular modeling studies show the 4-hydroxyl on the B ring of isoflavones to be the binding site for the ER [11, 12]. Phytoestrogen is a general definition that has been applied to any plant substance or metabolite that induces biological responses in vertebrates and can mimic or modulate the actions of endogenous estrogens usually by binding to ERs [13].

Galstyan AM et al. studied in detail the chemical composition of Teucrium polium, growing in the territory of Armenia. It has been found that the main biologically active compounds of Tecrium polium are phenylpropanoid glycosides-verbascoside, poliumoside, teupolioside (up to 6 %). Flavonoid (up to 3 %) and phenylpropanoid (up to 6 %) glycosides are found in hydroponic Teucrium polium [14]. Some of them have been reported to have phytoestrogen-like activity and was a prerequisite that address the hypothesis of possible neuroprotective efficacy in OVX. We have previously shown that hydroponic Teucrium polium has a protective effect on spike activity flow in cholinergic neurons in the nucleus basalis of Meynert following bilateral OVX [15].

The current study aimed to assess the neuroprotective effects of hydroponic Teucrium polium on hippocampal electrophysiological parameters (high frequency stimulation induced neuronal activity) and morpho-histochemistry (revealing of the activity of Ca^2+^-dependent acid phosphatase) in bilaterally OVX rats.

## Methods

### Animal models

Adult female albino rats weighing 250 ± 20 g were purchased from the experimental center of Orbeli Institute of Physiology. The animals were maintained at 25 ± 2 °C and 12 h light – dark cycle, lights on 07:00–19:00 h. The animals were provided food and water ad libitum. All of the experimental protocols were approved by the Committee of Ethics of the Yerevan State Medical University (YSMU) (Yerevan, Armenia), followed the “Principles of laboratory animal care” and were carried out in accordance with the European Communities Council Directive of 24 November 1986 (86/609/EEC).

OVX was performed on 6 months-old anesthetized (Pentobarbital 35 mg/kg, intraperitoneal) albino rats. A small abdominal incision was made. The ovaries were than located, and a silk thread was tightly tied around the oviduct, including the ovarian blood vessels. The oviduct was sectioned and the ovary removed. The skin and muscle wall were then sutured with silk thread.

Rats were divided into 4 groups: i) sham-operated (sham), ii) sham + Teucrium polium, iii) ovariectomized (OVX), iv) OVX + Teucrium polium. Starting from 4th week OVX + Teucrium polium (*n* = 7) and sham + Teucrium polium (*n* = 5) groups received i/m injection of Teucrium polium (20 mg/kg, 0.5 ml for 3 weeks). Starting from 4th week following OVX, both sham (*n* = 7) and OVX (*n* = 7) group rats were treated with 0.5 ml of distilled water.

### Teucrium polium injection and preparing the plant extract

Teucrium polium used in this study was collected from G.S. Davtyan Institute of Hydroponics Problems NAS RA. Teucrium polium is grown under hydroponic conditions and experimentally was shown that hydroponically grown plants have higher productivity yield. Hydroponic plant was harvested from August to October 2014 from G.S. Davtyan Institute of Hydroponics Problems NAS RA, which is the well-known production site of Teucrium polium in Armenia. The plant was botanically authenticated and voucher specimens were deposited in the Herbarium of Institute of Hydroponics (outdoor hydroponics, Experimental Hydroponic Station). For the study were obtained the 50 % ethyl alcohol soluble extracts of wild, hydroponic and soil Teucrium polium and the extract was then dried with rotary vacuum evaporator and were separated benzene, ethyl acetate, chloroform-methanol (3:1) and aqueous fractions. Hydroponic Teucrium polium is less toxic and has active enough ingredients and therapeutic investigation was carried out with water fraction of ethanol extract (related to the flavonoid glycosides and phenolic glycosides in the fraction). Aqueous fraction of ethanol extract of hydroponic Teucrium polium is considered as therapeutic (intramuscularly, 0.5 ml) [14, 16, 17]. 5 % from maximum endurable dose, equal to 400 mg/kg (equivalent to 20 mg/kg) of hydroponic Teucrium polium, refers to therapeutic dose [17] and for injection prepared daily and then water fraction of ethanol extract of hydroponic Teucrium polium diluted in sterile distilled water and single doses of hydroponic Teucrium polium were injected intramuscularly (0.5 ml) into the rats.

### In vivo electrophysiology and data analysis

**Fig. 1:**
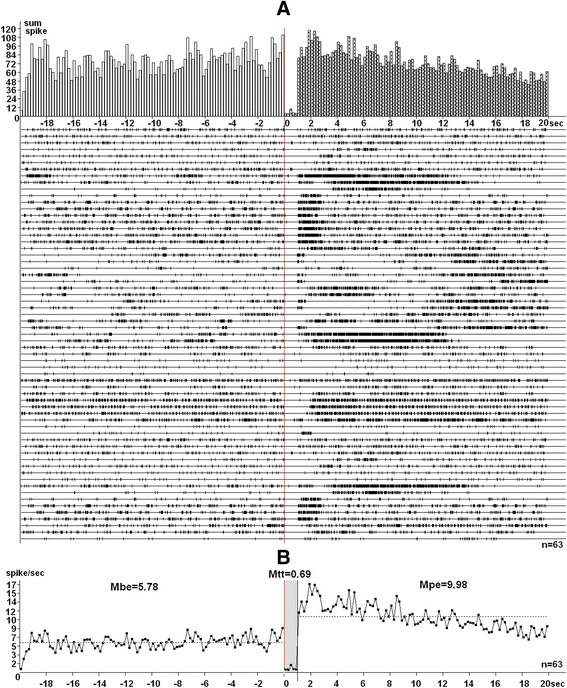
**a** – peristimulus histograms for the sum of spikes based on the analysis of spike activity of hippocampal neurons (presented as a raster) with TD-PTP type of responses, recorded in response to HFS of entorhinal cortex, **b** – diagrams of average spike frequency present mean values of frequency (spike/sec) of neuronal spike flow in real time 20 s before HFS (Mbe), 20 s after HFS (Mpe) and 1 s during HFS (Mtt); n - number of neurons used to calculate the averaged frequency of spiking activity for the diagram

The animals were anesthetized (Urethan 1.1 g/kg), immobilized with 1 % ditiline (25 mg/kg i/p), fixed in a stereotaxic head frame and were placed on artificial ventilation. The sample of isolated rat brain was obtained by transection of spinal cord (T2 – T3). The stimulatory electrode was inserted in the ipsilateral entorhinal cortex (EC) according to stereotaxic coordinates [18] (AP – 9, L ±3.5, DV +4.0 mm) and a glass recording electrode (1–2 μm tip diameter) filled with 2 M NaCl was repeatedly submerged into the hippocampal field CA1, CA3 at coordinates (AP − 3.2–3,5; L ± 1.5–3.5; DV +2.8–4.0 mm) for recording spikу activity flow of single neurons. High frequency stimulation (HFS) (100 Hz for 1 s) was performed by means of rectangle charge of 0.05 ms duration and 0.08–0.16 mA amplitude. Recording and mathematical analysis of spiking activity were carried out on the basis of the program (worked by V.S Kamenetski) providing selection of spikes by amplitude discrimination, which pinpoints spikes and excludes artifacts during HFS, allowing not only posttetanic, but also tetanic activity evalution [19]. The timing, frequency and cumulative histograms, as well as a diagram of mean frequency for single neurons and populations of neurons with uniform responses were constructed on the basis of analysis of peristimulus spiking. The aim of the analysis was to determine the statistical significance of differences for spike frequency before and after action of HFS and during HFS. To further evaluate, neuronal units and spike flow of those have significance levels of 0.05, 0.01, 0.001 were selected. Tetanic potentiation (TP) or tetanic depression (TD) and following posttetanic potentiation (PTP) and posttetanic depression (PTD) were recorded to HFS ipsilateral EC (Fig. 1).

### Morphohistochemistry

Hippocampus was fixed for 2–3 days in 5 % neutral formalin prepared on phosphate buffer (pH 7.4) at 4 ° C for 48 h. The frontal frozen sections were 50–60 μm. They were washed in distilled water and transferred to an incubation mixture for the selective detection of nerve cells: 20 ml of 0.38 % lead acetate solution and 5 ml 1 M acetate buffer (pH 5.6), 5 ml of 2 % solution of b-glycerophosphate sodium solution. Incubation was performed in a thermostat at 37 ° C for 1–3 h. This was followed by washing in distilled water, sections, film development in the sodium sulfate solution and, after repeated washing the samples were ready. The sections were processed using the new approach of detecting the activity of Ca^2+^-dependent acid phosphatase (AP) developed by I.B. Meliksetyan [19, 20]. In analogous experimental conditions and on the same animals the histochemical study of hippocampus was carried out after the electrophysiological studies. This methodical approach is based on detection of intracellular phosphorus-containing substances playing the key role in the metabolic energetic processes aimed at preservation and self-reproduction of vital systems. Apart from its histochemical significance, this method is of a certain morphological interest. The resulting picture yields greatly significant information, and allows critical evaluation of the specific chains of metabolism of the studied structures. Using the transmitted light background of preparation, the nervous structures are revealed clearly and are permanently reproduced, which is an important criterion of reliability of the method’s fidelity. The aforementioned was the basis for using the method of detection of orthophosphates in this investigation for study of the morphofunctional status of the cellular brain structures.

### Statistical analysis

For statistical evaluation we used t-criteria of Student’s t -test, the reliability of differences of interspike intervals before, after and during HFS. To increase reliability of statistical evaluations, we also used the non-parametric method of verification by application of Wilcoxon two-sample test.

## Results

### Electrophysiological study

After 8 weeks, in the sham, OVX and OVX + Teucrium polium groups, microelectrophysiological investigations of extracellular registration of background and induced spike activity of single neurons of hippocampus under HFS of EC were performed.

**Fig. 2:**
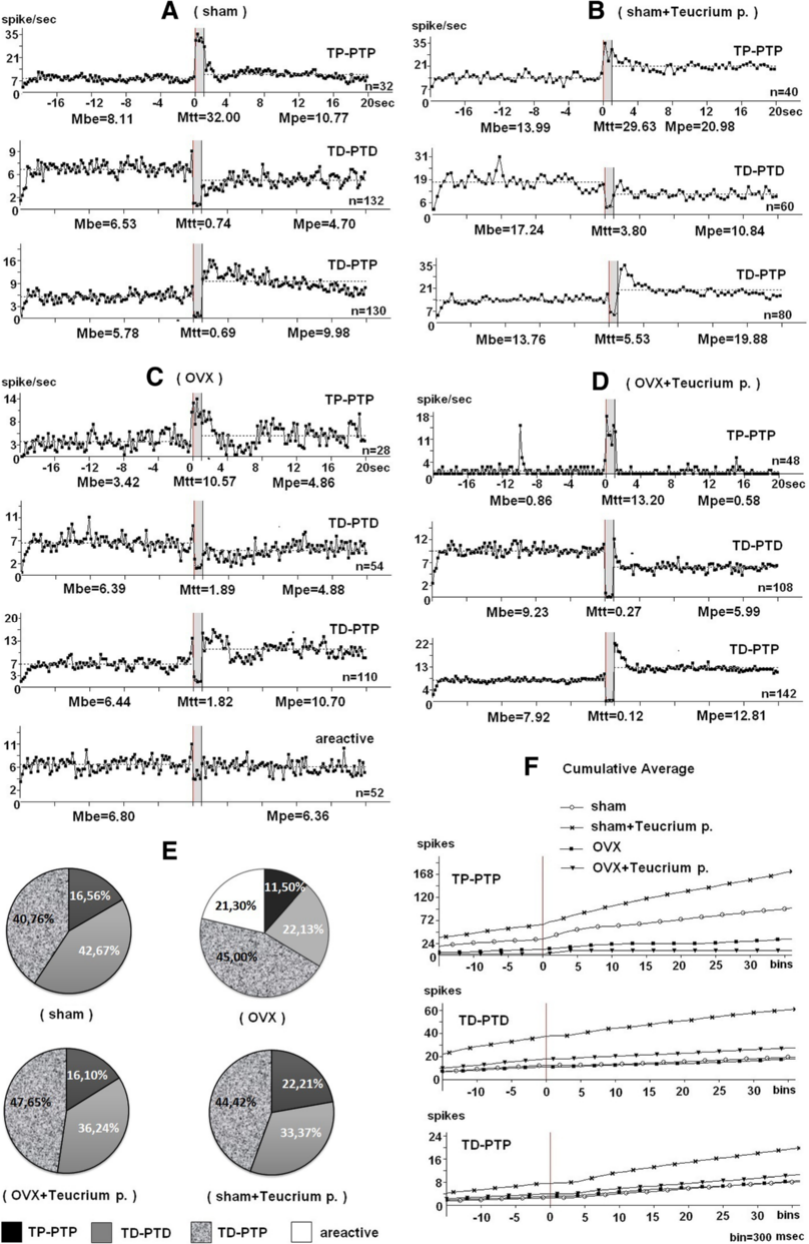
Diagrams of average spike frequency are presented with mean values of frequency (spike/sec) for TP-PTP, TD-PTD, TD-PTP type of neuronal response in real time 20 s before HFS (Mbe), 20s after HFS (Mpe) including HFS (Mtt) of entorhinal cortex for sham (**a**), sham + Teucrium polium (**b**), OVX (**c**), OVX + Teucrium polium (**d**) experimental groups; n - number of neurons used to calculate the averaged frequency of spiking activity for the diagram. **e** – proportions of response type (%) in hippocampal neurons in mentioned experimental groups. **f** – comparative averaged cumulative curves for TP-PTP, TD-PTD, TD-PTP types of responses

In rats in the sham group, responses in hippocampal neurons (*n* = 294) during HFS of EC were recorded in the form of TP-PTP (*n* = 32), TD-PTD (*n* = 132) and TD-PTP (*n* = 130). Diagram of mean frequency of peristimulus spike activity of neurons with TP-PTP responses (Fig. 2a) indicates the 4-fold expressed excitatory response during HFS (32:8.11 spike/sec). According to mean frequency of population of neurons with TD-PTD responses (Fig. 2a), TD expressed 8.8 times (6.53:0.74) and PTD 1.4 times (6.53:4.70). In a population of neurons with TD-PTP responses (Fig. 2a) TD expressed 8.4 times (5.78:0.69) and PTP-1.7 times (9.98: 5.78).

In the OVX group, 244 hippocampal neurons during HFS of EC were recorded as follows: TP-PTP with 3.09 fold (10.57:3.42) TP (Fig. 2c); 3.5 fold (6.44:1.82) TD and 1.66 fold (10.70: 6.44) PTP in a population of neurons with TD-PTP responses (Fig. 2c); 3.38 fold (6.39:1.89) TD and 1.3 fold (6.39:4.88) PTD in population of neurons with TD-PTD responses (Fig. 2c). In this case, the maximum proportions were neurons with TD-PTP responses (110 of 244 units) and were characterized by areactive units (21.30 % or 52 of 244 units) (Fig. 2e).

After 8 weeks in OVX + Teucrium polium group neurons (*n* = 298) were characterized by the highest mean frequency background activity (M_be_ = 9,23) in neurons with TD-PTD responses (*n* = 108) (Fig. 2d); 15.3 fold (13.20:0.86) TP in neurons with TP-PTP responses (*n* = 48) (Fig. 2d) and 1.6 fold (12.81:7.92) PTP in a population of neurons with TD-PTP responses (*n* = 142) (Fig. 2d). TD was expressed 34 fold (9.23:0.27) in a population of neurons with TD-PTD responses and 66 fold (7.92:0.12) in neurons with TD-PTP responses (Fig. 2d). A comparison of expression of cumulative curves of peristimulus spike activity in populations of neurons with TP-PTP, TD-PTD and TD-PTP was carried out on the basis of software analysis averaged for all compared sham, sham + Teucrium, OVX, OVX + Teucrium polium groups (Fig. 2f). It was found that 1) neurons in OVX + Teucrium polium group with TP-PTP responses have a lower level of peristimulus spike activity compared with those in sham and OVX groups, 2) in OVX + Teucrium polium group level of spike activity of hippocampal neurons exhibiting TD-PTD responses exceeds those in OVX and sham, 3) hippocampal neurons with TD-PTP responses have the same level in sham, OVX and OVX + Teucrium polium groups.

In the sham group during HFS EC in certain hippocampal neurons were recorded following balance of types of responses: TP, PTP, TP + PTP (16.56 %); TD, PTD, TD-PTD (42.67 %) and TD-PTP (40.76 %) (Fig. 2e). In OVX group 21.30 % of recorded neurons showed areactivity during HFS EC, while in sham, sham + Teucrium polium and OVX + Teucrium polium groups, those were absent (Fig. 2e).

In the sham + Teucrium polium group, in hippocampal neurons (*n* = 180) of intact animals treated with Teucrium polium intramuscularly for 3 weeks spike activity was recorded by following balance of components of responses: TP-PTP (22, 21 %), TD-PTD (33.37 %), TD-PTP (44,42 %) (Fig. 2e).

For this group of neurons with TP-PTP, responses exhibited 2.11 times expressed TP (29,63:13,99) and neurons with TD-PTD responses by exhibited 4.5 times expressed TD (17.24: 3.80). Dominated neurons with TD-PTP responses during HFS EC respond by 2.49 fold inhibition (13.76:5.53) and 1.44 fold excitation (19,88:13,76) (Fig. 2b). All recorded neurons of sham + Teucrium polium group were characterized by the highest mean frequency background and poststimulus activity (Fig. 2b).

### Morphohistochemical study

**Fig. 3:**
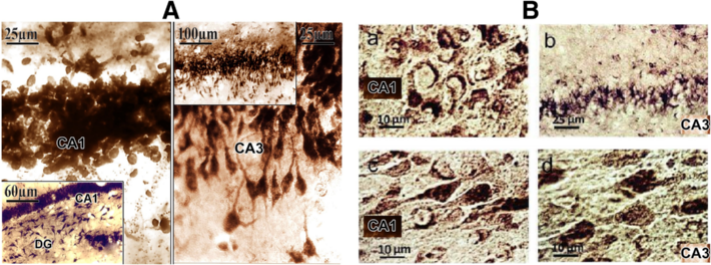
The frontal hippocampal slices of hippocampal sham (**a**), OVX rats (**b**-a, b, hippocampal CA1 and CA3 fields) and treated with hydroponic Teucrium polium (**b**-c, d). Neurons of hippocampal CA1, CA3 fields of the rat injected i/m with the extract of hydroponic Teucrium polium. a – picture of the “splitted” neuron; b – partly absence of neuronal reaction; c – restoration of normal morphology neuron, the restoration of the apical dendrites with high activity of acid phosphatase d – recovery of the form, sizes and enzyme activity of hippocampal neurons

After 8 weeks of OVX in hippocampal CA1, CA3 fields, the neurons were characterized by rounding and swelling of cell body compared with sham (Fig. 3a, b). The morphological pattern was characterized by a sharp drop in phosphatase activity in fields CA1, CA3. Hippocampal neurons have a structured violation; pyramidal cells completely lose the reaction of neurofibrillary. In the field CA1 and CA3, there is rounding and swelling of neuronal soma, the bodies of neurons were going round and swelling in the CA3, most of which were subject to chromatolysis, but nucleus of neurons occupied the central disposition and degenerative changes were also observed. After i/m injection of hydroponic Teucrium polium the sizes and forms of the cells are recovered (Fig. 3c), the enzyme activity was increased and efficiently intensified leading to cellular survival, positive changes in neuronal structures and enhanced metabolism. There was a marked increase in the density of cells in hippocampal CA1, CA3 fields (Fig. 3c, d).

## Discussion

Earlier Galstyan A.M. et al. showed that hydroponic Teucrium polium contains flavonoid, terpenoid, iridoid and phenilpropanoid glycosides, exhibiting high biological activity [21, 22]. The expediency of cultivation of Teucrium polium in open hydroponics, in order to obtain the ecologically clean yield and programmed chemical composition-enrichment by flavonoid glycosides (apigenin, luteolin) and phenolic glycosides (verbascoside, poliumoside, teupolioside) was established [23]. It was shown that Teucrium polium L (due to its proven AChE inhibitory capacity and antioxidant properties) is a new promising candidate and alternative medicine for treatment or prevention of Alzheimer’s disease and related disorders [24, 25]. On the other hand, flavonoids, which found in plants simulate some hormones and neurotransmitters, have been shown to scavenge free radicals [26]. Antiacetylcholinesterase and antioxidant activities [27] as well as high acetylcholinesterase (AChE) and butyrylcholinesterase (BChE) inhibitory activities of ethanol extract of Teucrium polium have also been reported [28]. Teucrium polium (high in flavonoids) has been suggested due to its promising pharmacological profile [29]. The effect of flavonoid luteolin on long-term potentiation (LTP) and memory occurred by activation of CREB (cAMP response element-binding), which supports the therapeutic potential of luteolin for synaptic function [30]. Apigenin exerts modulatory effects on GABAergic and glutamatergic transmission in cultured cortical neurons [31]. In hippocampal cells, apigenin inhibited kainic acid-induced excitotoxicity in a dose-dependent manner and showed neuroprotective effects [32]. Regarding to antioxidant, anticholinesterase and hypoglycemic effects, Teucrium polium reversed learning and memory deficits of diabetic rats [33]. The results of our study indicate that the activity of the synaptic apparatus under the influence of Teucrium polium recovered compared with the characteristic of degenerative neurons the depletion of spiking in OVX group. A significant increase in expression of excitatory and depressor responses during HFS in OVX + Teucrium polium group (compared with sham group) promotes the organization of network activity in the new conditions of compensatory adaptations. Reduced synaptic activity (compared to the sham), apparently compensated by increase in the share/number of neurons with excitatory type of responses in neural circuits of EC-hippocampus. The weakening of neurons to response during HFS indicates disturbance of neuromediation and spike activity impairment following OVX and, conversely, a certain stimulus frequency reproduction is an indicator of recovery of neurotransmitter status (whereupon areactive neurons were not detected) in OVX-Teucrium polium group. In this regard, it was shown that phenylethanoid glycosides are able to attenuate the glutamate-induced neurotoxicity at concentrations ranging from 0.1 to 10 microM [34]. It was found that activity-guided fractionation of Teucrium chamaedrys and Nepeta cataria led to the isolation of the caffeoyl phenylethanoid glycosides teucrioside, verbascoside and lamiuside A (teupolioside), which have a direct interaction with calcineurin [35]. Calcineurin is one of the most abundant protein phosphatases in the nervous system and acts on multiple substrates in synaptic, cytoplasmic and nuclear compartments in neuronal cells. Dysregulation of calcineurin in the diseased brain is one of the major causes of pathological Ca^2+^signaling associated with cognitive disorders [36]. Calcineurin selectively enhances L-type Ca(2+) channel activity in hippocampal neurons [37]. The content of nitric oxide, the activity of nitric oxide synthase and the expression of caspase-3 protein were decreased by verbsacoside [38]. This has highlighted a compensatory, neuroprotective role for NO that protects synapses by increasing neuronal excitability (a potential mechanism for augmentation of excitability by nitric oxide is via modulation of voltage-gated potassium channel activity) [39].

Ca 2+ signalling is important dialogue between the synapse and cell nucleus and plays a vital role in cell survival and synaptic plasticity [40]. It was shown that in hippocampal neurons, CREB-dependent gene expression is linked to the long-lasting phase of activity-dependent neuroprotection [41]. The net effect of transcriptional changes includes limiting of executioner caspases, apoptosis inhibition and remaining of viability and electrical activity of neurons [42]. In morphofunctional aspect the regulation of nuclear architecture by synaptic and extrasynaptic NMDARs is of major importance: neurons translate NMDAR signals into changes in nuclear geometry, providing a means for an activity-dependent modulation of nucleo-cytoplasmic exchanges [43]. These modulator processes apparently play a significant role in the formation of morphofunctional parameters (restoration of normal morphology of neuron, the restoration of the apical and side dendrites with high activity of acid phosphatase) and exert protective effects of Teucrium polium during OVX-induced hippocampal neuronal degeneration.

## Conclusion

Thus, the results of in vivo electrophysiological and morphofunctional experiments regarding the recovery of functioning and morpho-histochemistry of hippocampal neurones showed the efficacy of Teucrium polium following OVX. With regard to the exact mechanisms of Teucrium polium’s action in animal OVX model, we suggest that hydroponic Teucrium polium may improve OVX-induced neuronal impairment through activation of ERs-mediated cell survival signaling. Our results suggest that Teucrium polium may exert a potential therapeutic value and point toward new approach to drug discovery for clinical therapies of menopause memory impairment.

## Abbreviations

AChE: Acetylcholinesterase
AChE: Acetylcholinesterase
BChE: Butyrylcholinesterase
CREB: cAMP response element-binding
E2: Estradiol
EC: Entorhinal cortex
ERα: Estrogen receptor alpha
ERβ: Estrogen receptor beta
GABA: Gamma-Aminobutyric Acid
HFS: High frequency stimulation
LTD: Long-term depression
LTP: Long-term potentiation
NMDAR: N-methyl-D-aspartate receptor
OVX: Ovariectomy
PTD: Posttetanic depression
PTP: Posttetanic potentiation
TD: Tetanic depression
TP: Tetanic potentiation
